# Application of Bayesian network and regression method in treatment cost prediction

**DOI:** 10.1186/s12911-021-01647-y

**Published:** 2021-10-16

**Authors:** Li-Li Tong, Jin-Bo Gu, Jing-Jiao Li, Guang-Xuan Liu, Shuo-Wei Jin, Ai-Yun Yan

**Affiliations:** 1 Cancer Hospital of China Medical University, Shenyang, China; 2 College of Information Science and Engineering, Northeastern University, Shenyang, China; 3 Liaoning Cancer Hospital & Institute, Shenyang, China

## Abstract

Charging according to disease is an important way to effectively promote the reform of medical insurance mechanism, reasonably allocate medical resources and reduce the burden of patients, and it is also an important direction of medical development at home and abroad. The cost forecast of single disease can not only find the potential influence and driving factors, but also estimate the active cost, and tell the management and reasonable allocation of medical resources. In this paper, a method of Bayesian network combined with regression analysis is proposed to predict the cost of treatment based on the patient's electronic medical record when the amount of data is small. Firstly, a set of text-based medical record data conversion method is established, and in the clustering method, the missing value interpolation is carried out by weighted method according to the distance, which completes the data preparation and processing for the realization of data prediction. Then, aiming at the problem of low prediction accuracy of traditional regression model, this paper establishes a prediction model combined with local weight regression method after Bayesian network interpretation and classification of patients' treatment process. Finally, the model is verified with the medical record data provided by the hospital, and the results show that the model has higher prediction accuracy.

## Introduction

In recent years, with the rapid growth of medical and health care expenditure, the public health system has also exposed some problems, such as uneven distribution of medical resources, unable to meet the growing medical needs and so on [1–3]. Through the patient diagnosis information research disease treatment cost prediction is an important way to promote the pricing mechanism reform, control the unreasonable growth of medical costs, reduce the burden of patients, and affect the main causes of treatment costs. Factors often include the patient's condition, treatment options and treatment cycle.

For a long time, the industry has carried out extensive and in-depth research on the prediction of medical expenses, and achieved remarkable results [4–7]. The existing methods can be divided into two categories: diagnosis based and data-based prediction. According to the historical treatment cost of a large number of the same patients, the data-based prediction method approximately predicts the possible cost of current patients through machine learning method. Wang et al. [8] studied the daily hospitalization number and medical expenses of patients with mental disorders from January 1, 2011 to December 31, 2015. Time series analysis was established to estimate the total annual health expenditure, hospitalization expenses and annual medical expenses of mental disorder patients. This study shows the long-term trend of total direct medical expenses for mental illness, and forecasts the results. Chen et al. [9] studied the influence of the number of health workers employed in public health care sector, the number of population and the number of Inpatients Per 100 people on the total health expenditure from 2003 to 2011 in Serbia Growth. By using statistical analysis and multiple linear regression analysis, the author concluded that the growth of health workers during this period strongly promoted the growth of total health expenditure. Data based methods are usually suitable for the situation of long time series and large amount of data. At this time, data statistical methods can better find the rules of variables and the relationship between variables. However, this also limits that when the amount of data is small or the data distribution does not have a long time span, the statistical methods often have large errors. The diagnosis method is based on the disease as the starting point, through mathematical methods to explain the pathological characteristics to achieve the purpose of prediction. Compared with the data-based method, this method is more targeted, and more suitable for the case of small amount of data or small number of diseases. Qing and Liu [10] proposed that it is necessary to pay more attention to the disease itself in the study. At the same time, four kinds of predictive variables were established in the study. By using multiple regression analysis and back propagation neural network, the factors influencing the medical expenses of single disease cataract were found out, and the acceptable medical expenses were predicted by two regression models. Kim and Park [11] used medical examination data, laboratory test, self-reported medical history and self-reported health behavior data to establish high-cost user prediction model by three methods: logical regression, random forest and neural network model, and determined the characteristics of medical examination as the prediction factor of high-cost users. This method mainly aims at the cost prediction of high-cost medical users, so the data set itself is a high-cost user, which not only limits the needs of ordinary users, but also can not see the role of ordinary users in the medical structure system. At present, diagnosis based research is mostly to single disease. And the amount of data is not very huge. One reason is that the method pays more attention to the law of the disease itself rather than the change of the value. The other reason is that the research methods used in the method are more regression analysis or simple neural network [9, 12–14]. Therefore, the diagnosis method is to establish a model to simulate the development of the disease and determine the diagnosis and treatment plan. At present, the commonly used regression methods are logistic regression, multiple linear regression and so on. These methods focus more on the relationship between independent variables and dependent variables, so they lack the cause and effect of the disease itself.

In this paper, we propose a Bayesian network and regression analysis method to predict the cost of treatment by using the diagnosis cases. Firstly, based on the patients' cases, the disease influencing variables are extracted. And the variable transformation and missing value processing are carried out. The redundant variables are eliminated through correlation analysis, and the data set of the model is obtained. Then, the Bayesian network is used to simulate and analyze the multi category disease description variables, and the patients are divided into different treatment schemes. The local weighted LASSO regression analysis is carried out under each treatment scheme, and a higher accuracy prediction model is obtained. Finally, the model is validated by the data of colon cancer patients in a hospital, and the prediction effect of the model is compared with that of regression model and neural network model. The results show that the proposed method can better simulate the diagnosis and treatment method selection, and its prediction accuracy is better than that of traditional regression method and neural network model.

## Data preprocessing

This paper intends to use mathematical thinking to deal with medical problems, so the selected variables should have a certain mathematical form on the premise of representing the patient's condition as much as possible. In this paper, we selected four kinds of disease information which can affect the cost, including patient's age, gender, surgery history, treatment plan, past pathology and disease condition, current admission condition, smoking condition, diabetes mellitus, hypertension, etc. They are: patient's explanation, history of present illness, medical history and personal history. Main complaints refers to the explanation of symptoms and personal information by patients or the question and answer between doctors and patients. History of present illness is the patient's present symptoms. It also includes the current treatment in the process of the disease. Medical history is that the patient had other operations and other chronic diseases before. Personal history represents the patient's living place, smoking, drinking and other information.

Most of these variables are written descriptions, which can not be used as formula variables for operation, so the first step in data processing is to digitize the text information. At the same time, because the case records are from different doctors, the format is not uniform, so there will be some missing variables. Therefore, in order to maximize the role of data, the missing variables should be deleted or the difference should be made according to their accuracy.

### Text variable digitization

There are many descriptive variables in text variables, such as patient's gender and medical history, which can not be directly used as model input. Moreover, it is difficult to establish a unified conversion standard for these variables due to the disease and other reasons. Therefore, this paper develops a set of text numerical method suitable for the current disease through mathematical thinking, which is verified by experts.

Electronic medical record contains not only numerical variables, but also some descriptive variables. In the model, numerical variables can be used for calculation, and descriptive variables also have an important impact on the prediction of the patient's condition. Therefore, this paper first establishes a unified standard for descriptive variables in medical records. After the analysis of descriptive variables in medical records, they can be divided into two categories. One is qualitative descriptive variable, the other is degree descriptive variable. Qualitative descriptive variables, such as abdominal pain, smoking, etc., are represented by a value of 0–1. Degree descriptive variables such as mild abdominal distension and recurrent hematochezia also need to be converted into numerical type. In this paper, all data sets are traversed and all degree descriptive variables are selected. In this paper, it is considered that the description first has the property characteristics, and then has the degree description. Therefore, in the numerical conversion of the variable, the characteristics of the variable should be taken into account. In this paper, firstly, the basic value is given according to the characteristics, then the severity is divided into different levels and scored according to different levels. Finally, the basic value and severity value are weighted to get the final variable value. The conversion function is as follows.

1 $$\left\{ {\begin{array}{*{20}l} {y = 0,\;\;b = 0} \hfill \\ {y = 0.5 + \frac{x}{2a},\;\;b \ne 0} \hfill \\ \end{array} } \right.$$

where *y* is the variable value after transformation, *b* is whether the patient has the symptom, 0 is not exist, non-0 is exist, *a* is the total number of the disease degree levels, and *x* is the degree level value of the patient.

The numerical method is based on the guidance of doctors and experts, and is compatible with the qualitative and severity characteristics of symptom feature description. It is a conversion method defined to infer the severity of disease. This method adopts the linear formula method, which can still maintain the discrete characteristics of discrete variables. It also unifies and standardizes the numerical format, which can standardize the numerical value and avoid unnecessary deviation of the model due to the span problem after data conversion.

### Missing data processing

After further analysis of the data, there is a problem of missing some features in the electronic medical record, because it is not from the same doctor at the time of recording, and there is no unified standard. For the problem of missing data processing, the commonly used method is to interpolate the global mean value [15]. This method will lead to the same interpolation of the same kind of variables, and there is a large error. In this paper, an improved method based on KNN (K-Nearest Neighbor) proximity method is proposed to calculate the missing value by weighting the distance between adjacent points.

The missing data in medical records are processed according to the missing proportion. First, check the data in the variable with serious missing information. Each medical record is set as an array X_i_ = [X_i1_, X_i2_, …, X_i(n+1)_]. It contains n characteristic variables and one target variable. Each array may contain some missing values. For some medical records, when the missing value exceeds 20%, there is no further consideration in the model, because too much difference will lead to inaccurate prediction information.

For the data whose missing value is less than 20%, the k nearest variable data is used, and the missing value is estimated by weighted evaluation according to the distance. If the common distance average method is used to estimate the missing value, there will be a large error, so according to the nearest point of the variable, and according to the distance to allocate the weight, the prediction error can be reduced. In this paper, the weight is learned from the overall data distribution, which contains all the information, and can also be combined with local information to get the final accurate distance estimation.

2 $$f(x) = \frac{{\sum\nolimits_{i = 1}^{k} {D_{i} W_{i} } }}{{\sum\nolimits_{i = 1}^{k} {W_{i} } }}$$

where *f*(*x*) is the distance from the test point to the cluster center, W_*i*_ stands for weight, D_*i*_ represents the distance between the nearest neighbor *i* and the test point.

The traditional difference method is based on the global average interpolation, the difference does not have individual differences, which is suitable for the large amount of data to ensure that it does not affect the trend of the interpolation method. When the number of cases is small, the error of the traditional method will be magnified, and the method in this paper can interpolate according to the data near the real variables, which ensures the rationality, but also has a certain degree of heterogeneity. The values computed by the proposed method can be closer to the real value in the verification experiment.

## Bayesian and regression fusion method

The core of the fusion method of Bayesian network and regression analysis is to classify first and then regress, so as to predict the treatment cost of patients on the basis of describing the treatment process of patients. Due to the small amount of case data, and the large difference of drug cost and dosage between different treatment schemes, if all data are used for regression analysis, the error is large. Therefore, this paper does not use the statistical method, but uses the Bayesian network which can make full use of the characteristics of the disease.

### Bayesian network

Bayesian network is a directed acyclic graph composed of nodes and a group of conditional probability tables between nodes. The graph model is mainly composed of two parts: the network topology of nodes and the conditional probability table of nodes [16].

The generation of Bayesian network mainly includes two parts: one is to determine the dependence between variables, that is, to determine the network structure; the other is to determine the conditional probability between variables, that is, the weight of network nodes. The difficulty of determining the network structure is to traverse all the network structures, so the variables should be independent of each other, and the relationship between nodes should follow the Bayesian principle.

In order to classify the case variables, this paper analyzes the relationship between features based on features. The variable *x* = (*x*_1_, *x*_2_, …, *x*_*n*_) is defined, which has n features extracted from cases, and the class *y* = (*y*_1_, *y*_2_, …, *y*_*j*_) refers to the J classifications extracted from cases. The purpose of Bayesian network is to explain the reason of category y with variable x. According to Bayesian principle, the formula is as follows:

3 $$P\left({y}_{j}|{x}_{1},{x}_{2},\dots ,{x}_{n}\right)=\frac{P({y}_{j}){\mathrm{P}}({x}_{1},{x}_{2},\dots ,{x}_{n}|{y}_{j})}{{\mathrm{P}}({x}_{1},{x}_{2},\dots ,{x}_{n})}$$

4 $$\widehat{{y}_{j}}={\mathrm{argmax}}P\left({y}_{j}\right)\prod_{i=1}^{n}{\mathrm{P}}\left({x}_{i}|{y}_{j}\right)$$

where $P\left({y}_{j}|{x}_{1},{x}_{2},\dots ,{x}_{n}\right)$ is the conditional probability that a pair of variables is assigned to no category, $P({y}_{j})$ is the probability value of each class, $\widehat{{y}_{j}}$ is the number of categories of variable X.

Based on the obtained patient feature information, each feature is discrete and independent of each other. In order to apply it to Bayesian network, the characteristic variable X is used to explain the reason of variable y after removing the high correlation. Variable X interprets class y, which is a mathematical interpretation method for patient diagnosis. The directed probability value of each variable is a process of restoring facts through mathematical methods. Therefore, in the process of training, we need to ensure the rationality of the network through multiple groups of cross validation.

The maximum likelihood estimation is a statistical method based on the maximum likelihood principle, which can be simply described as: suppose that a random trial has multiple possible results A, B, C, etc. If result A appears in a random trial, that is to say, the possibility of result a is very large, it can be considered that the test conditions are favorable for result a. The maximum likelihood estimation of Bayesian network is to calculate the value of a given parent node set, take the frequency of different values of each node as the conditional probability parameter of the node, and try to find the parameter that maximizes the likelihood function of the node.

Because the maximum point of the log likelihood function is consistent with the maximum point of the likelihood function, and the calculation is more convenient, the log likelihood function is often used to replace the likelihood function. The formula is as follows:

5 $$l\left(H|E\right)={\mathrm{logL}}(H|E)$$

where H and E are two random variables and L is likelihood function.

Bayesian network model can judge the treatment plan selected by patients through the case data. This part can not only provide suggestions for patients to choose treatment plan, but also make a certain classification for further regression analysis, so as to reduce the prediction error caused by the category difference of the data itself.

### Local weighted LASSO regression

With the continuous development and improvement, the theory of multiple linear regression is relatively mature. It can find out the quantitative relationship between variables, describe the law of numerical change between statistical variables, and finally predict. It is an effective way to accurately learn the influence degree and direction of independent variables on dependent variables.

The linear regression equation describes how the dependent variable *y* depends on the independent variable and the error value *ε*. The equation can be written as follows:

6 $$y= {\beta }_{0}+{\beta }_{1}{x}_{1}+{\beta }_{2}{x}_{2}+\cdots +{\beta }_{k}{x}_{k}+\varepsilon$$

Among them, ${\beta }_{0}$ is the regression constant, ${\beta }_{1,}{\beta }_{2}\dots ,{\beta }_{k}$ is the regression coefficient, ${x}_{1},{x}_{2},\dots ,{x}_{k}$ is the regression variable and *ε* is the error term.

In order to fit the data features in the model training, the error term in the model can be set as a variable. Its role is to reduce the impact of the number of data. In solving mathematical equations, the number of variables should not be greater than the number of conditions. Therefore, when the amount of data is not large enough, classification will cause the solution of the system to become unstable. Therefore, the function of the error term is to simplify the model and improve the generalization ability by setting the coefficients of some low action variables to 0 in the iterative process. In the experimental comparison model, LASSO linear regression method performs better in generalization ability. Therefore, in the setting of error term, the iteration can be set to order 1, and the iteration formula is as follows:

7 $$\varepsilon = \min \frac{1}{m}\left[ {\lambda \sum\limits_{i = 1}^{m} {|\beta_{i} |} } \right]$$

where *m* is the number of samples, λ is the regularization coefficient, ${\beta }_{j}$ is the model parameter.

At the same time, an upper limit should be made for the coefficient of error term, so as to compress the coefficient of low action variable and simplify the parameters of the model. The qualification is as follows:

8 $$\sum\limits_{i = 1}^{n} {|\beta_{i} |} \le \sigma$$

when the value of *σ* is modified, the variable *β* will be amplified or compressed. When the variable *β* is compressed to a minimum, some variables will be infinitely close to 0. This variable can be regarded as a low action variable, and the action of this variable can also be regarded as 0. Therefore, this method can simplify the model structure and accurately fit the model when there are few variables.

A local weighting method is proposed. The weight function is as follows. The selection of weight function is based on the normal distribution of data distribution, so the iterative weight coefficient can be expressed as follows:

9 $${\omega }^{(i)}={\mathrm{exp}}\left(-\frac{{x}^{(i)}-x}{2{\sigma }^{2}}\right)$$

In model training, variable X presents normal distribution characteristics according to the target variable value, so each time in model training, the weight can be adjusted according to the data distribution position, and the adjustment function is as above. In this method, the adjustment function is applied to the regular term, and the adjusted regular term is as follows:

10 $$\varepsilon = \min \frac{1}{m}\left[ {\lambda \sum\limits_{i = 1}^{m} {\omega^{(i)} \beta_{i} } } \right]$$

Because the random data often presents the normal distribution law, the data in this paper also has this characteristic after verification, so the local weighting method proposed in this paper can further highlight the role of important data, and can improve the prediction accuracy of the model on the premise of ensuring the complexity of the model.

## Example analysis

In this paper, 240 cases of patients in the Department of Enterology in a hospital in Shenyang in March 2016 were selected as the validation data. After sample analysis and preprocessing, the prediction model of Bayesian network and regression analysis fusion was established, and the accuracy of the model was verified with the data obtained from preprocessing.

### Sample data analysis

**Fig. 1:**
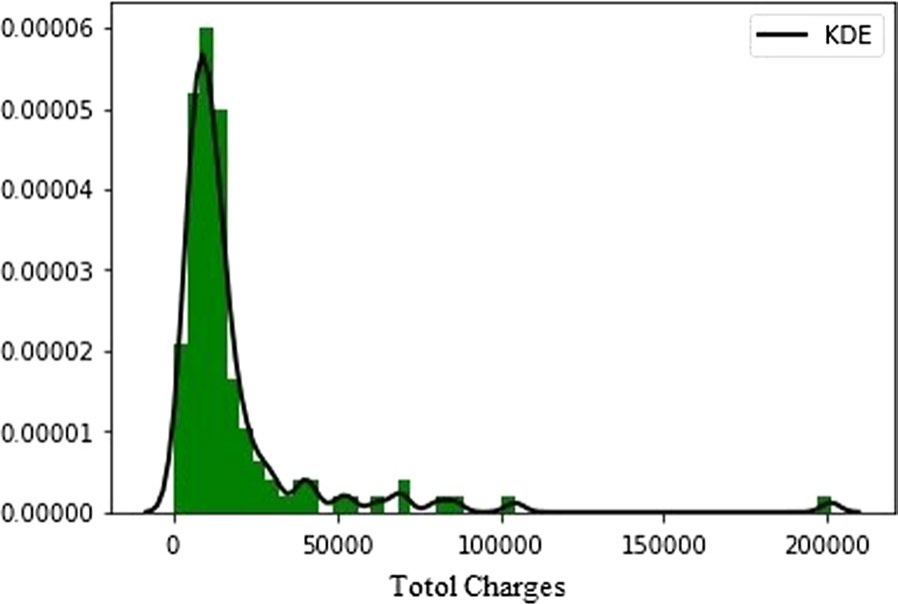
Data distribution characteristics. Figure shows the cost distribution of case data. It can intuitively show the law of data distribution, so as to provide some ideas for the improvement of our model. Kernel Density Estimation (KDE) is a method to study the data distribution characteristics from the data sample itself

Through the preliminary analysis of the sample, the sample shows a normal distribution, without a large number of abnormal data, and the data set itself can be used for model validation. The sample distribution is shown in Fig. 1.

Each medical record consists of four parts, including patient's explanation, history of present illness, medical history and personal history. Each type of variable contains multiple sub variables. The total number of variables selected by the model is 26. Because some variables are highly correlated, LASSO model is used to select and 16 variables are left. The patient's explanation included gender A1, age A2, postoperative time A3 of rectal cancer, history of present illness including urethral symptoms B1, defecation symptoms C2, pathology B3, cancer metastasis B4, radiotherapy B5, chemotherapy B6, fever B7, medication B8, previous history including diabetes C1, hypertension C2, other surgical history C3, personal history including smoking D1, drinking D2.

In cross validation, discrete random variables are selected as validation variables, which have a large distribution range and randomness. Here we select the variable age.

It can be seen from the table that the interpolation method in this paper can reduce the interpolation error to a certain extent compared with the traditional method, which is particularly important for the prediction results in the case of small amount of data.

**Fig. 2:**
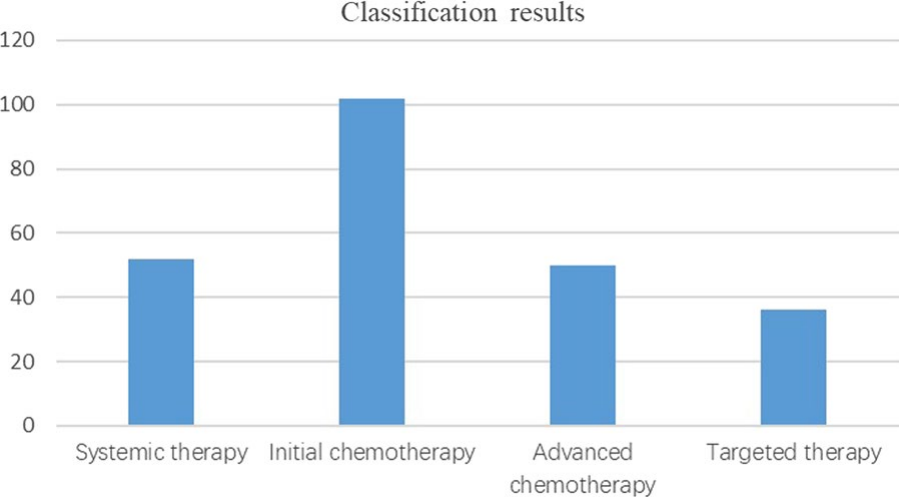
Distribution of treatment schemes. Figure shows the distribution of treatment regimens. This distribution can also reflect the distribution law of treatment plan of patients at this stage. In order to give a more detailed treatment plan and the prediction of the treatment plan to provide a certain reference

The intermediate variable is the result of Bayesian network classification. According to the treatment plan, it can be divided into four categories: systematic treatment, primary chemotherapy, secondary chemotherapy and targeted therapy. The data distribution is shown in Fig. 2.

### Analysis of prediction results

**Fig. 3:**
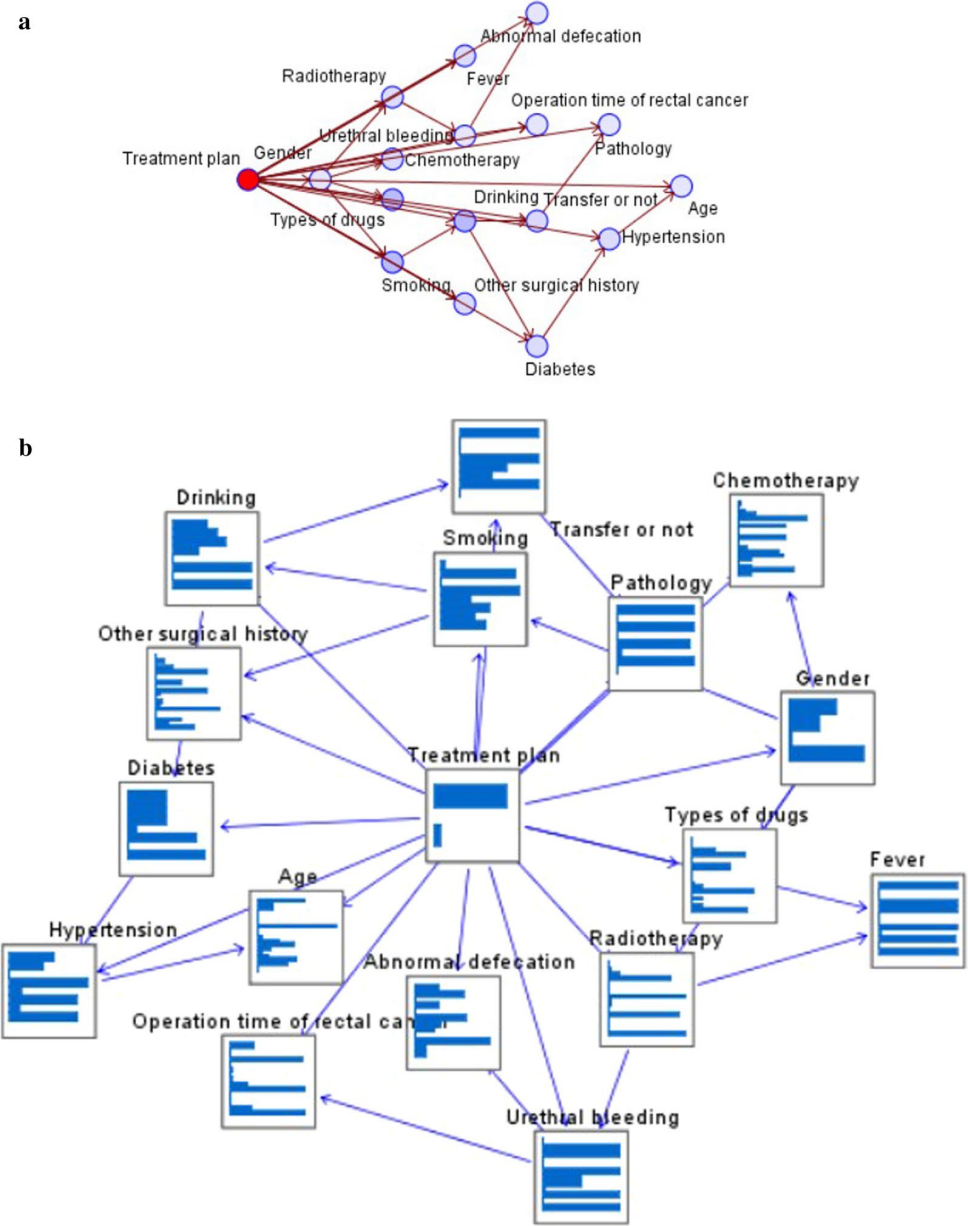
Bayesian classification structure. Figure is the result graph of Bayesian network. **a** Mainly shows the network structure of Bayesian network, and mainly experiences the structure and hierarchical relationship of directed graph. **b** Mainly shows the probability relationship of each node's corresponding event. The node variable names are Gender, Age, Operation time of rectal cancer, Abnormal defecation, Urethral bleeding, Pathology, Transfer or not, Radiotherapy, Chemotherapy, Fever, Diabetes, Hypertension, Other surgical history, Smoking, Drinking, Types of drugs, Treatment plan

Input the processed data into the Bayesian network structure, this paper obtains the analysis structure diagram of each variable to the treatment scheme, and obtains the probability value of each node variable, and the structure is shown in Fig. 3.

In Fig. 3, the model is divided into seven layers. The first layer is the predicted target variable treatment plan. The second level is an important basic variable gender. In the result analysis, gender is an important influencing variable, which will have an important impact on the follow-up third level chemotherapy, radiotherapy, drug types and smoking. The fourth level is the upper level variables related to fever, urethral bleeding, alcoholism and other surgical history. From the relationship between the two variables, we can see that alcoholism has a certain tendency to smoking. The fifth layer is abnormal defecation, operation time of rectal cancer, metastasis and diabetes. The sixth layer is pathology and hypertension. In the relationship between hypertension and diabetes, hypertension has a certain tendency to diabetes. The seventh level is age, which shows that age is the most basic variable (Table 1).

After classifying the data for treatment scheme selection, the data will be regressed and fitted for each category to obtain the prediction accuracy of Bayesian network and LASSO regression model, and the accuracy of Bayesian network and LASSO regression model will be compared with linear regression, traditional LASSO regression and neural network model. The indexes of evaluation results are accuracy, mean square error (MSE) and R-Square (R2), as shown in Table 2 below. MSE is the average of the square of the difference between the predicted value and the true value. The range of R-squared is 0–1. The larger it is, the better the model fitting effect is.

**Table 1:** Cross validation of interpolation methods [17]

Method	Deviation rate (%)	Prediction accuracy of linear regression model (%)	Forecast standard deviation
Global average method	6.44	56.15	5.14
Fixed value method	8.95	54.92	8.31
Local KNN method	3.21	59.74	2.94

Table mainly shows the interpolation error and its influence on the prediction results in different interpolation methods. The advantages of this method are shown

**Table 2:** Comparison of accuracy of model prediction

Model	Accuracy (%)	MSE	R2
Linear regression model	59.74	13.32	0.58
LASSO regression model	65.78	10.88	0.66
Neural network model	63.45	11.06	0.62
Locally weighted LASSO regression model	85.65	6.38	0.75
Bayesian network fusion local weighted LASSO regression model	89.14	5.36	0.81

Table shows the accuracy, mean square error (MSE) and R-Square (R2) of the prediction results obtained by various regression and prediction methods. MSE is the average of the square of the difference between the predicted value and the true value. The range of R-squared is 0–1. The larger it is, the better the model fitting effect is

It can be seen from the above table that the prediction accuracy of traditional linear regression model is low. The main reason is that the model is too complex and the generalization ability is poor due to too many independent variables. Therefore, in the traditional LASSO model, the coefficient of independent variable with low effect is set to 0, which greatly simplifies the complexity of the model and slightly increases the accuracy of the model. Neural network model has strong self-learning and adaptive ability, so the prediction accuracy of this model is better than linear regression model, but slightly lower than LASSO regression model. Therefore, in order to further optimize the prediction accuracy of regression model, regression analysis is carried out on the basis of Bayesian network classification. The prediction accuracy of the final model is improved to 89.14%. And it is 23.36% percentage points higher than that of the traditional LASSO regression model. The model optimization effect is significant. And the prediction accuracy is still 3.49% percentage points higher than that of the same regression model without classification. At the same time, the model can select the treatment plan for patients, and can provide more suitable plan for patients in practical application.

## Conclusion

Based on the statistics and analysis of the patient electronic medical record, the characteristics of the patients’ condition are extracted. Then distance weight is added to KNN method to estimate the missing value. Then, the cases were classified, and the classification results were modeled by the local weighted regression method. The prediction model of patients' treatment cost was successfully established, which was suitable for the situation of low amount of data and aimed at improving the prediction accuracy. Using the data provided by the hospital to verify the model, it is found that this method has higher accuracy than the traditional method, reaching 89.14%, 23.36% higher than LASSO regression model with the best effect in the comparison model, and 3.49% higher than the same regression model without classification. Based on the prediction of treatment cost, this paper can also recommend the treatment options for patients, and it is also the key to further improve the accuracy of the same method.

The establishment of the model provides a certain reference for the prediction of medical related expenses, and the processing of text medical records also provides a feasible method for the text to be used in data analysis. The next possible work will be to further improve the accuracy of classification, or reduce the prediction error in the case of wrong classification.

## Data Availability

Not applicable.
